# Balancing Genome Plasticity and Evolutionary Constraint During the Long-Term Evolution of Feline Coronaviruses

**DOI:** 10.3390/v18091037

**Published:** 2026-09-19

**Authors:** Xuejia Wen, Qilin Zhao, Wenqiang Wang

**Affiliations:** 1 Jiangsu Co-Innovation Center for Prevention and Control of Important Animal Infectious Diseases and Zoonoses, Institute of Comparative Medicine, College of Veterinary Medicine, Yangzhou University, Yangzhou 225009, China; 2 Jiangsu Key Laboratory of Zoonosis, Jiangsu Interdisciplinary Center for Zoonoses and Biosafety, Yangzhou University, Yangzhou 225009, China

**Keywords:** feline coronavirus, recombination, positive selection, CpG depletion, genome plasticity, evolutionary constraint

## Abstract

Feline coronaviruses (FCoVs) are evolutionarily dynamic alphacoronaviruses whose diversification has been driven by extensive genetic exchange and long-term adaptation. However, how genome plasticity is balanced with the evolutionary constraints required to maintain viral fitness remains poorly understood. Here, we analyzed 127 complete FCoV genomes collected worldwide over more than five decades to investigate the evolutionary processes governing long-term FCoV diversification. Genome-wide phylogenetic analyses resolved three major evolutionary lineages and demonstrated that the two recombinant lineages originated independently through recombination between feline and canine coronaviruses, highlighting repeated interspecies recombination as a recurrent source of genomic innovation. In contrast, gene-level evolutionary analyses revealed substantial discordance with whole-genome phylogenies, indicating that individual genomic regions have followed distinct evolutionary trajectories. Recombination and positive selection were concentrated within a limited subset of genes, particularly NSP3, NSP12, NSP2, and the spike gene, suggesting that evolutionary innovation preferentially targets proteins involved in virus–host interactions. Despite extensive genome restructuring and adaptive diversification, overall dinucleotide composition remained highly conserved across all lineages, with persistent CpG suppression and continued CpG depletion during the long-term evolution of the predominant GI lineage, revealing strong compositional constraints acting on FCoV genomes. Together, these findings support a multi-scale model of coronavirus evolution in which recombination reorganizes genome architecture, adaptive evolution refines genes at the virus–host interface, and long-term genomic constraints preserve fundamental compositional features. This work provides a conceptual framework for understanding how genome plasticity and evolutionary constraint are balanced during the long-term evolution of FCoVs and offers broader insights into the evolutionary organization of alphacoronavirus genomes.

## 1. Introduction

Coronaviruses are among the most genetically diverse RNA viruses, infecting a broad range of mammalian and avian hosts. Their evolutionary success is largely attributed to a combination of genomic plasticity and adaptive flexibility, enabled by large RNA genomes, complex replication mechanisms, and frequent genetic exchange [1]. Unlike many RNA viruses whose diversification is primarily driven by nucleotide substitution, coronaviruses can acquire substantial genetic variation through recombination, allowing the generation of novel genomic architectures and the emergence of distinct evolutionary lineages [2]. Understanding how recombination-driven innovation is balanced by functional and host-associated evolutionary constraints is essential for elucidating the mechanisms underlying coronavirus diversification and adaptation.

Alphacoronaviruses represent a particularly dynamic lineage within the coronavirus family, with numerous examples of host-associated diversification and interspecies genetic exchange [3,4]. Feline coronaviruses (FCoVs) are globally distributed alphacoronaviruses that provide an important model for studying viral evolution within long-term host populations. FCoVs circulate extensively among domestic and wild felids worldwide and exhibit remarkable genetic diversity despite their relatively limited host range [5]. Although most infections are asymptomatic or associated with mild enteric disease, evolutionary changes accumulated during persistent circulation can occasionally contribute to the emergence of feline infectious peritonitis virus (FIPV), a pathogenic variant responsible for fatal systemic disease [6]. While substantial progress has been made in understanding the molecular determinants associated with FCoV pathogenic transition, the evolutionary processes shaping FCoV diversity at the population level remain less well defined.

A prominent feature of FCoV evolution is its close evolutionary relationship with canine coronaviruses (CCoVs) [7]. The emergence of type II FCoVs has been attributed to historical recombination events involving CCoVs, particularly affecting the spike gene, providing a well-characterized example of cross-species genetic exchange within alphacoronaviruses [8]. Both FCoVs and CCoVs have been reported to utilize aminopeptidase N (APN/CD13) as a functional receptor, although receptor usage can vary according to viral type and host species [9,10,11]. This shared receptor usage provides a plausible biological context for cross-species compatibility of spike proteins, but the specific host and epidemiological circumstances in which FCoV–CCoV recombination occurred remain unknown. This evolutionary history highlights the capacity of recombination to introduce foreign genomic material and generate new viral lineages. However, coronavirus genomes are not simply collections of interchangeable genetic segments. Recombined regions must function within new genomic contexts and remain subject to constraints imposed by protein function, replication requirements, and host interactions. Therefore, the evolutionary consequences of recombination depend not only on the generation of genetic diversity but also on subsequent selection and genomic compatibility.

Despite increasing availability of complete FCoV genome sequences, the evolutionary processes governing FCoV diversification remain incompletely understood. Previous studies have primarily focused on specific genomic regions, particularly the spike gene, because of its central role in receptor recognition, host adaptation, and pathogenicity [12]. However, a genome-wide perspective is required to determine how different regions of the FCoV genome contribute to lineage emergence and long-term diversification. In particular, understanding whether FCoV evolution is dominated by episodic acquisition of novel genomic components or by continuous adaptation within established lineages remains an important unresolved question.

Viral evolution is shaped by multiple interacting forces beyond recombination [13]. During long-term circulation within hosts, viruses accumulate genetic changes while remaining constrained by selective pressures imposed by viral biology and host immunity. These evolutionary constraints can influence genome composition, coding sequence variation, and the distribution of adaptive changes across viral genes. Such processes are especially relevant for coronaviruses, whose large genomes encode proteins with diverse functions ranging from replication and genome maintenance to host interaction and immune modulation. Consequently, different genomic regions may follow distinct evolutionary trajectories, with some regions exhibiting extensive diversification while others remain evolutionarily constrained [14].

FCoVs therefore provide an opportunity to investigate how genomic innovation and evolutionary constraints interact to shape coronavirus diversity over extended evolutionary timescales [7,15]. Although individual recombinant FCoV strains and specific recombination events have been described in previous studies, these observations have largely been considered in the context of particular strains, genomic regions, or geographic populations. A broader genome-wide assessment is therefore needed to determine how such individual evolutionary events collectively contribute to the long-term diversification of FCoVs. Such an approach can reveal whether genetic exchange, nucleotide-level adaptation, and selective forces act independently or collectively to define the evolutionary landscape of circulating FCoVs.

In this study, we analyzed 127 complete FCoV genomes collected globally over more than five decades to investigate the evolutionary mechanisms underlying FCoV diversification. Rather than focusing on the identification of individual evolutionary events, we integrated phylogenetic reconstruction, recombination analysis, gene-level evolutionary comparisons, genome composition analysis, and selection assessment to examine how multiple evolutionary processes jointly shape FCoV diversification across genomic scales. These findings provide insights into the evolutionary dynamics of alphacoronaviruses and improve our understanding of how coronavirus genomes diversify while maintaining functional integrity.

## 2. Materials and Methods

### 2.1. Sequence Collection and Dataset Preparation

Complete genome sequences of feline coronaviruses were retrieved from the NCBI GenBank database using combinations of relevant keywords, including “feline coronavirus” and “feline infectious peritonitis virus”. The retrieved sequences were screened based on their associated metadata and genomic annotations to construct a dataset representing naturally occurring FCoVs. Only sequences annotated as complete or near-complete genomes with sufficient sequence integrity for whole-genome evolutionary analysis were retained. Sequences with substantial missing regions or incomplete genome coverage were excluded. Sequences identified as laboratory-derived, synthetic, engineered, chimeric, vaccine-associated, or otherwise artificially generated viruses were excluded. After quality control and manual curation, 127 complete FCoV genomes were retained for subsequent evolutionary analyses. The accession numbers of all 127 genomes are provided in Supplementary Table S1. The final dataset represented viruses sampled from multiple geographic regions and spanned more than five decades, thereby providing broad temporal and geographic coverage of FCoV diversity.

### 2.2. Multiple Sequence Alignment and Phylogenetic Analyses

The 127 complete FCoV genome sequences were aligned using MAFFT (v7.518) with the automatic algorithm selection strategy [16]. The resulting multiple-sequence alignment was subsequently trimmed using trimAl (v1.5.0) with a gap threshold of 0.05. Alignment columns in which gaps were present in more than 95% of the sequences were excluded from the alignment [17]. The resulting alignment was used for maximum-likelihood phylogenetic reconstruction.

A maximum-likelihood (ML) phylogenetic tree was reconstructed using IQ-TREE 2 [18]. For the whole-genome phylogenetic analysis, the optimal nucleotide substitution model was determined using the integrated ModelFinder module based on the Bayesian information criterion (BIC), which identified UNREST + FO + R5 as the best-fitting model. The ML tree was subsequently inferred under this model, and branch support was assessed using 1000 bootstrap replicates.

### 2.3. Recombination Analyses

Recombination was investigated at both the whole-genome and individual-gene levels. Whole-genome analysis was performed to characterize potential mosaic genomic structures and identify putative recombination breakpoints, whereas gene-level analyses were conducted to assess the distribution of recombination signals across the FCoV genome.

Potential recombination events at the whole-genome level were investigated using SimPlot (v3.5.1) [19]. Representative FCoV genomes from the GIIa and GIIb lineages, including PQ192636.1 and PQ133177.1, respectively, were compared with candidate parental genomes representing FCoVs from GI and canine coronaviruses. Candidate parental sequences were selected based on their phylogenetic relationships and sequence similarity to the query genomes.

Pairwise nucleotide sequence similarity between each query genome and candidate parental sequences was calculated across the complete genome using a sliding-window approach. A window size of 500 nucleotides and a step size of 50 nucleotides were applied. Similarity profiles were examined along the genome to identify changes in the relative similarity of query sequences to different candidate parental genomes. Abrupt transitions in the predominant similarity pattern were used to identify putative recombination regions and estimate the approximate locations of recombination breakpoints. The inferred recombinant regions were subsequently mapped onto the genome annotation to determine the corresponding genomic and coding regions.

Gene-specific recombination analyses were performed independently for all 24 annotated FCoV genes. For each gene, the corresponding nucleotide sequences were extracted from all 127 complete FCoV genomes, generating 24 independent gene-specific datasets, each containing 127 sequences. Each gene-specific dataset was independently aligned using MAFFT before recombination analyses.

The resulting alignments were analyzed using the Recombination Detection Program version 5 (RDP5) [20]. Seven complementary recombination detection methods implemented in RDP5 were applied, including RDP, GENECONV, BootScan, MaxChi, Chimaera, SiScan, and 3Seq. Default settings were used unless otherwise specified. To increase the robustness of recombination detection and reduce the influence of method-specific false-positive signals, a stringent consensus criterion was applied, whereby only recombination events supported by at least five of the seven methods were considered reliable. This criterion required concordant support from the majority of the independent detection methods rather than relying on a single algorithm. For each gene, sequences identified as recombinant according to this criterion were recorded, and the number of recombination-positive sequences was calculated to characterize the gene-specific distribution of recombination across the FCoV genome.

The numbers of recombination-positive sequences identified for the 24 genes were summarized and visualized as a heatmap using the ggplot2 package in R (v4.3.1). The resulting heatmap was used to compare the relative distribution of recombination-positive sequences among individual FCoV genes.

To independently evaluate whether each gene alignment contained statistically significant evidence of recombination, the same 24 gene-specific alignments were analyzed using the Pairwise Homoplasy Index (PHI) test implemented in SplitsTree (v6.3.30) [21]. The PHI test assesses incompatibility among nucleotide sites and provides a statistical test for recombination signals within aligned sequence datasets. A *p* value < 0.05 was considered statistically significant and interpreted as evidence of detectable recombination signals within the corresponding gene. The PHI test results were evaluated alongside the RDP5 results to provide complementary evidence for gene-specific recombination across the FCoV genome.

### 2.4. Haplotype Network Analyses

Haplotype analyses were performed for the membrane (M) and nonstructural protein 10 (NSP10) genes to characterize gene-specific genetic diversity and evolutionary relationships among FCoVs. These two genes were selected because neither showed detectable evidence of recombination in the gene-level recombination analyses, thereby providing nonrecombinant genomic regions for assessing evolutionary relationships based primarily on nucleotide variation.

For each gene, unique nucleotide haplotypes were identified from the resulting alignments using DnaSP (v6.12.03), and the frequency of each haplotype was calculated based on the number of sequences carrying the corresponding nucleotide pattern [22]. The identified haplotypes were subsequently imported into PopART (v1.7) for network construction.

Haplotype relationships were inferred using the Neighbor-Joining (NJ) method implemented in PopART. The resulting haplotype networks were visualized to represent genetic relationships among unique haplotypes, with each node corresponding to an individual haplotype and connections reflecting their relative genetic divergence. Haplotype attributes were annotated according to the whole-genome phylogenetic lineages (GI, GIIa, and GIIb) to examine the distribution and genetic relationships of haplotypes across the major FCoV lineages.

### 2.5. Dinucleotide Composition and CpG Relative Abundance

Dinucleotide composition was characterized across the 127 complete FCoV genomes to assess nucleotide usage patterns and the relative abundance of dinucleotide pairs. For each genome, the frequencies of all 16 possible dinucleotides were calculated using the compseq program implemented in the EMBOSS (v6.6.0) suite. The word size was set to 2, and the observed and expected frequencies of each dinucleotide were calculated based on the nucleotide composition of the corresponding genome.

The relative abundance of each dinucleotide was quantified using the observed-to-expected (O/E) ratio, calculated as the observed frequency divided by the expected frequency. An O/E ratio of 1 indicates that the observed frequency is consistent with the expected frequency based on the underlying mononucleotide composition, whereas values below or above 1 indicate relative depletion or enrichment, respectively. The O/E ratios of all 16 dinucleotides were calculated independently for each genome and subsequently compared among the GI, GIIa, and GIIb lineages to characterize lineage-specific patterns and within-lineage variation in dinucleotide composition.

Because CpG exhibited the strongest relative depletion across the analyzed genomes, its temporal dynamics were further examined. The CpG O/E ratio for each genome was paired with its corresponding sampling year. Pearson correlation analysis was performed using the ggscatterstats function in the ggstatsplot package in R to assess the association between CpG relative abundance and sampling year. The Pearson correlation coefficient (r) and corresponding *p* value were used to quantify the strength and statistical significance of the temporal association. Temporal correlation analysis was performed only for lineages with sufficient variation in sampling years.

### 2.6. Selection Pressure Analyses

Selection pressures acting on FCoV protein-coding genes were evaluated using the complete set of 127 FCoV genomes included in this study after sequence curation. Coding sequences corresponding to each of the 24 annotated FCoV genes were extracted from the complete genomes, and each gene was independently aligned at the codon level to preserve the reading frame and ensure accurate inference of synonymous and nonsynonymous substitutions.

The resulting gene-specific codon alignments were analyzed using the Fast Unconstrained Bayesian AppRoximation (FUBAR) method implemented on the Datamonkey web platform [23]. FUBAR was used to identify individual codon sites subject to pervasive positive or negative selection by estimating site-specific synonymous and nonsynonymous substitution rates under a Bayesian framework. Codon sites were considered positively selected according to the statistical criteria implemented by FUBAR. The positively selected sites identified across the 24 protein-coding genes were subsequently summarized and compared among genes to characterize the distribution of selective pressures across the FCoV genome.

## 3. Results

### 3.1. Evolutionary Relationships and Phylogenetic Diversity of FCoVs

**Figure 1:**
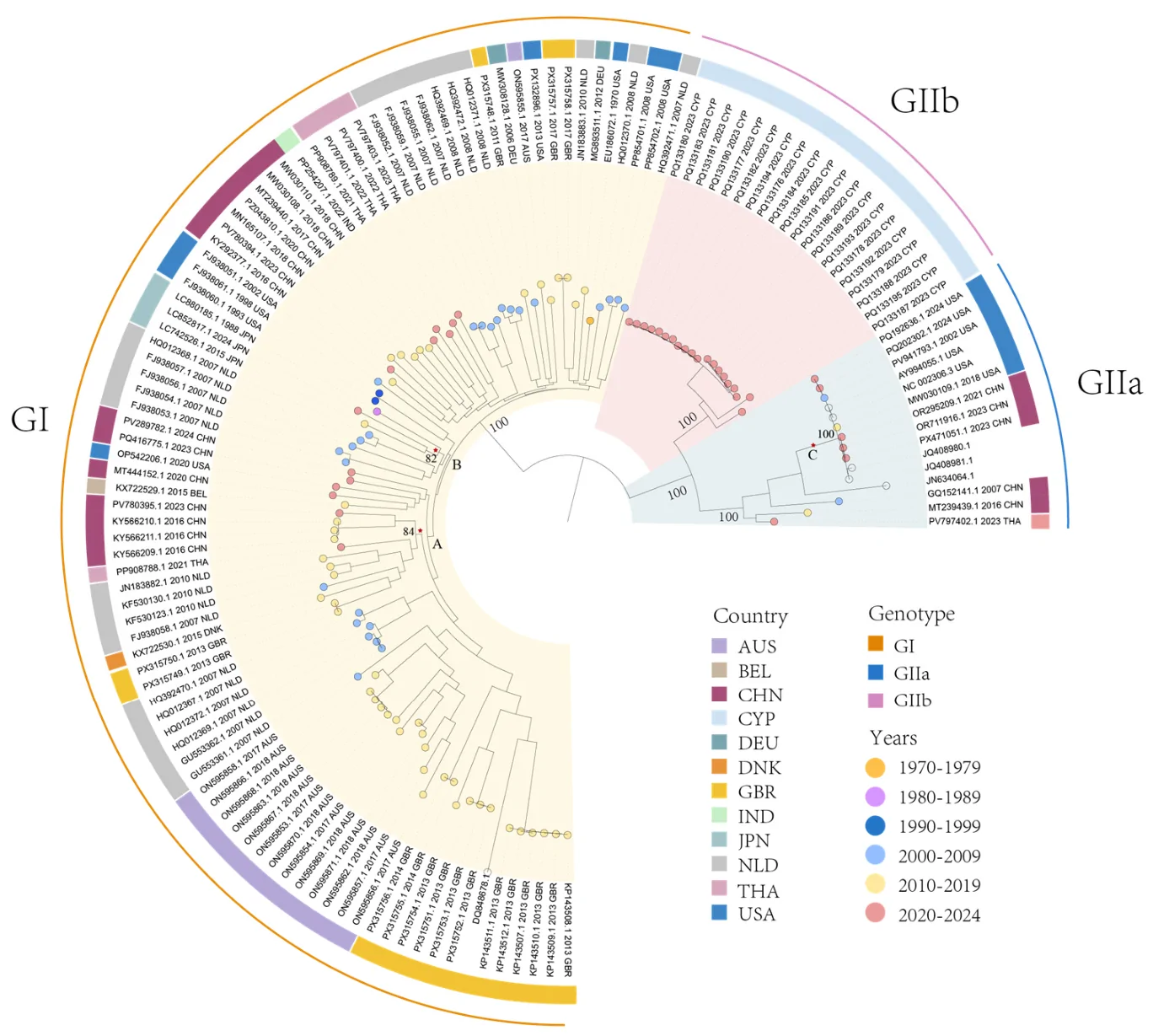
Global phylogenetic relationships and genetic diversity of feline coronaviruses. Maximum-likelihood phylogenetic tree reconstructed from 127 complete FCoV genomes. The three major evolutionary lineages, GI, GIIa, and GIIb, are indicated by the outermost annotation track. Terminal nodes are marked with colored circles according to the sampling year of each virus, while the outer concentric track indicates the geographic origin of the corresponding strains using distinct colors for different countries. Selected representative clades are labeled with letters. Bootstrap support values are shown at key internal nodes.

To investigate the evolutionary relationships among feline coronaviruses, a maximum likelihood phylogenetic tree was reconstructed using 127 complete genome sequences. The resulting phylogeny resolved three well-supported clades, each receiving a bootstrap value of 100, demonstrating the robustness of the inferred evolutionary relationships (Figure 1). These three clusters were designated GI, GIIa, and GIIb. The GIIa and GIIb lineages clustered together to form a strongly supported monophyletic group with a bootstrap value of 100, indicating that they share a more recent common ancestor with each other than with GI (Figure 1).

GI contained the largest number of FCoV genomes and included strains collected between 1970 and 2023. The phylogenetic topology revealed no apparent geographic clustering within this lineage, suggesting the absence of obvious geographic structure (Figure 1). Genetically distinct viruses were identified within the same region during similar sampling periods. For example, viruses circulating in Australia around 2017 were distributed across different positions within GI, with one cluster located in the upper portion of the tree and additional Australian strains occupying more basal branches (Figure 1). This pattern highlights the considerable genetic diversity of FCoVs circulating within the same geographic region.

Within GI, clade A comprised closely related strains originating from China, Thailand, the Netherlands, Denmark, and the United Kingdom, indicating that genetically similar FCoVs have undergone extensive international dissemination (Figure 1). This observation is consistent with the widespread circulation of FCoVs across Asia, Europe, Australia, and the Americas. In contrast, clade B consisted of three Japanese strains collected in 1988, 2015, and 2024, respectively (Figure 1). These viruses formed a well-supported monophyletic cluster, suggesting that closely related viral lineages can be maintained within a single geographic region over several decades. Overall, these findings demonstrate that the GI lineage has undergone a complex evolutionary history, with evidence of both widespread geographic dissemination and sustained local diversification, resulting in substantial genetic heterogeneity across time and space.

GIIa primarily comprised FCoV strains from the United States and China that were collected between 2002 and 2024. Within this lineage, clade C represented the predominant cluster and was characterized by extremely short terminal branches, indicating limited genetic divergence among circulating strains (Figure 1). Despite this high degree of genetic similarity, members of clade C have been detected across multiple countries over a period exceeding two decades, suggesting sustained circulation with relatively limited evolutionary diversification.

GIIb formed the sister lineage of GIIa and predominantly consisted of strains collected in Cyprus in 2023, including FCoV 23 (Figure 1). Similar to clade C of GIIa, most GIIb viruses also exhibited very short terminal branches, reflecting a high level of genomic conservation within the lineage. The highly similar phylogenetic topology observed within GIIb is consistent with the dissemination of closely related recombinant lineages rather than repeated independent recombination events.

### 3.2. Recombination Shapes the Genomic Diversity and Evolutionary History of FCoVs

To investigate the contribution of recombination to FCoV evolution, recombination analyses were conducted at both the whole genome and individual gene levels. Representative genomes from GIIa and GIIb were selected to characterize their recombination patterns.

**Figure 2:**
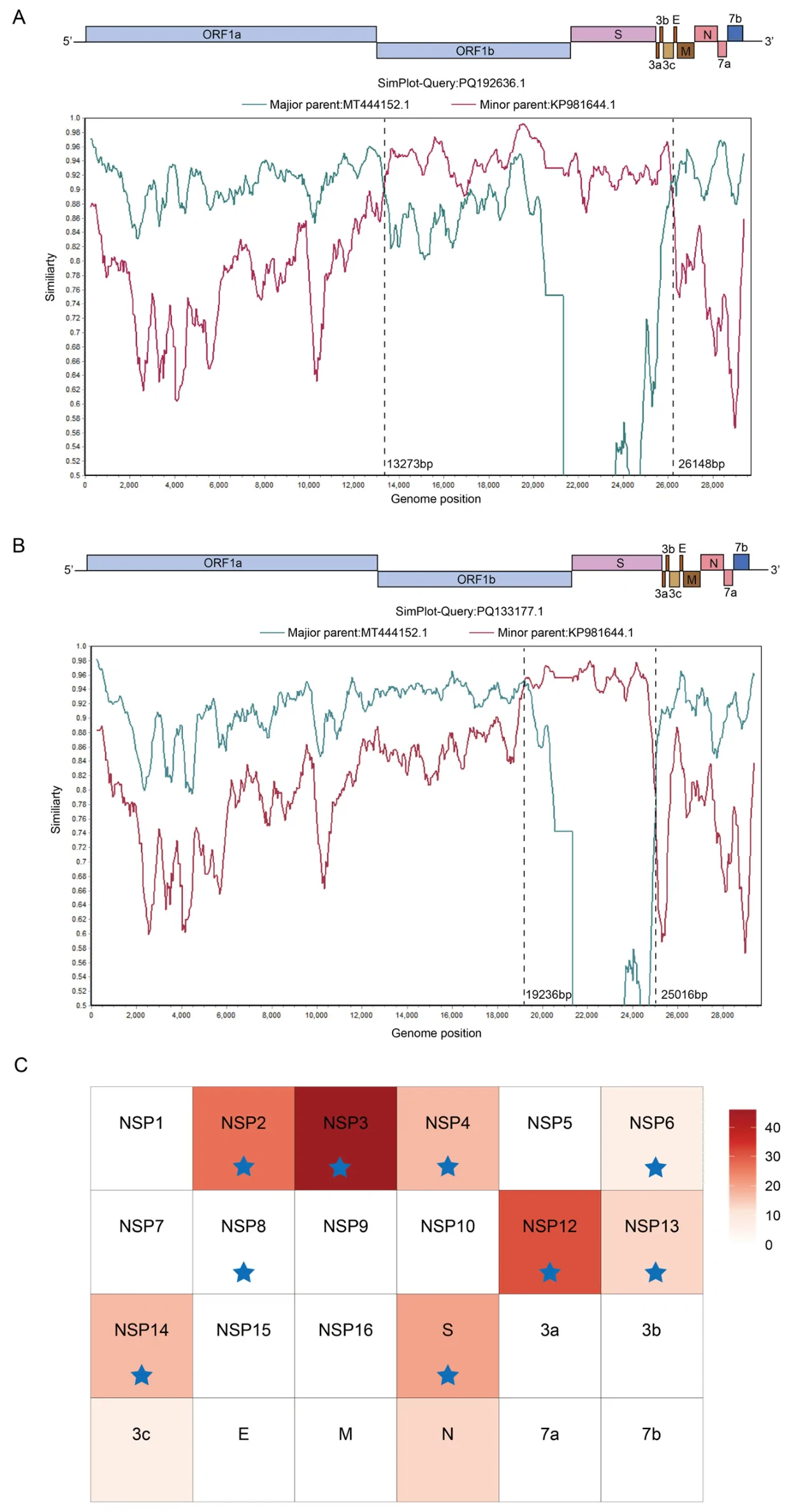
Genome-wide and gene-specific recombination patterns in feline coronaviruses. (**A**) SimPlot analysis of the representative GIIa genome PQ192636.1 against representative GI FCoV and CCoV parental sequences. Similarity profiles were generated using a 500 nt sliding window with a 50 nt step size. Putative recombination breakpoints are indicated by vertical dashed lines, and the genomic organization of FCoV is shown above the plot to indicate the positions of the corresponding coding regions. (**B**) SimPlot analysis of the representative GIIb genome PQ133177.1 against representative GI FCoV and CCoV parental sequences. Vertical dashed lines indicate the inferred recombination breakpoints, with the corresponding genomic organization shown above the plot. (**C**) Heatmap showing the distribution of recombination-positive sequences across the 24 FCoV genes. Each cell represents the number of sequences showing a reliable recombination signal for the corresponding gene, with color intensity proportional to the number of recombination-positive sequences. Recombination signals were considered reliable when supported by at least five of the seven recombination detection methods implemented in RDP5. The analyses were performed independently for each gene using all 127 FCoV genomes, with darker shading indicating a greater number of sequences exhibiting reliable recombination signals. Blue stars indicate genes showing significant recombination signals detected by SplitsTree.

For GIIa, SimPlot analysis identified PQ192636.1 as a recombinant genome generated through recombination between a GI FCoV and a canine coronavirus (Figure 2A). Most of the genomic backbone was inherited from the GI FCoV lineage, whereas the majority of ORF1b together with the complete spike gene and accessory genes 3a, 3b, and 3c originated from canine coronavirus (Figure 2A). Although an extensive genomic region was acquired from canine coronavirus, sequence similarity remained relatively high across most genomic regions, while the spike gene exhibited substantially greater divergence than the remaining coding sequences. This pattern indicates that the spike gene has accumulated considerably greater genetic variation than other genomic regions during the evolutionary history of these alphacoronaviruses.

A distinct recombination pattern was identified in GIIb. SimPlot analysis showed that PQ133177.1 also originated through recombination between a GI FCoV and a canine coronavirus, although the recombination breakpoints differed from those observed in GIIa (Figure 2B). In this genome, the major parental backbone was likewise derived from GI FCoV, whereas most of the spike gene together with the terminal region of ORF1b encoding NSP15 and NSP16 was contributed by canine coronavirus (Figure 2B). The contrasting recombination architectures observed in GIIa and GIIb indicate that these two lineages most likely originated from independent recombination events involving canine coronavirus rather than from a common recombinant ancestor.

Notably, the spike gene was involved in the recombination events that gave rise to both GIIa and GIIb, whereas the principal distinction between the two lineages was the genomic origin of ORF1b (Figure 2). This observation is consistent with the phylogenetic relationship in which GIIa and GIIb form closely related sister lineages while retaining distinct genomic architectures generated through different recombination histories (Figure 1). Collectively, these findings demonstrate that repeated recombination between FCoVs and canine coronaviruses has played a pivotal role in generating the genomic diversity of G2 lineages while maintaining their close evolutionary relationship.

To further characterize the recombination landscape across the FCoV genome, all 24 viral genes were independently analyzed using SplitsTree together with the seven recombination detection algorithms implemented in RDP5. Only recombination events supported by at least five of the seven RDP5 methods were considered reliable. The combined analyses revealed that recombination was widespread throughout the FCoV genome and affected both structural and nonstructural genes, although the extent of recombination varied markedly among individual genes.

Among all genes, NSP3 exhibited the highest frequency of recombination, with 46 recombinant sequences identified, followed by NSP12 with 31 sequences, NSP2 with 27 sequences, and the spike gene with 20 sequences (Figure 2C). Consistent with these findings, SplitsTree analyses of these genes displayed extensive reticulate network structures, providing independent evidence for frequent recombination and complex evolutionary relationships (Figure 2C).

In contrast, recombination signals were unevenly distributed across the genome. No evidence of recombination was detected in NSP1, NSP5, NSP7, NSP10, or the membrane gene by either RDP5 or SplitsTree, indicating that recombination has not contributed equally to the evolutionary history of all FCoV genes (Figure 2C). Instead, recombination exhibited pronounced gene specific variation, with several genes undergoing frequent recombination, whereas others remained highly conserved throughout their evolutionary history.

Taken together, these findings reveal a hierarchical pattern of recombination in FCoVs, whereby recurrent exchange of large genomic fragments contributes to lineage emergence, whereas frequent recombination within specific genes further accelerates sequence diversification during viral evolution.

### 3.3. Haplotype Network Analyses Reveal Gene Specific Evolutionary Patterns in FCoVs

To further investigate the contribution of mutation to FCoV evolution, haplotype network analyses were performed for the M and NSP10 genes, representing structural and non-structural genes, respectively, both of which showed no detectable evidence of recombination (Figure 2C).

**Figure 3:**
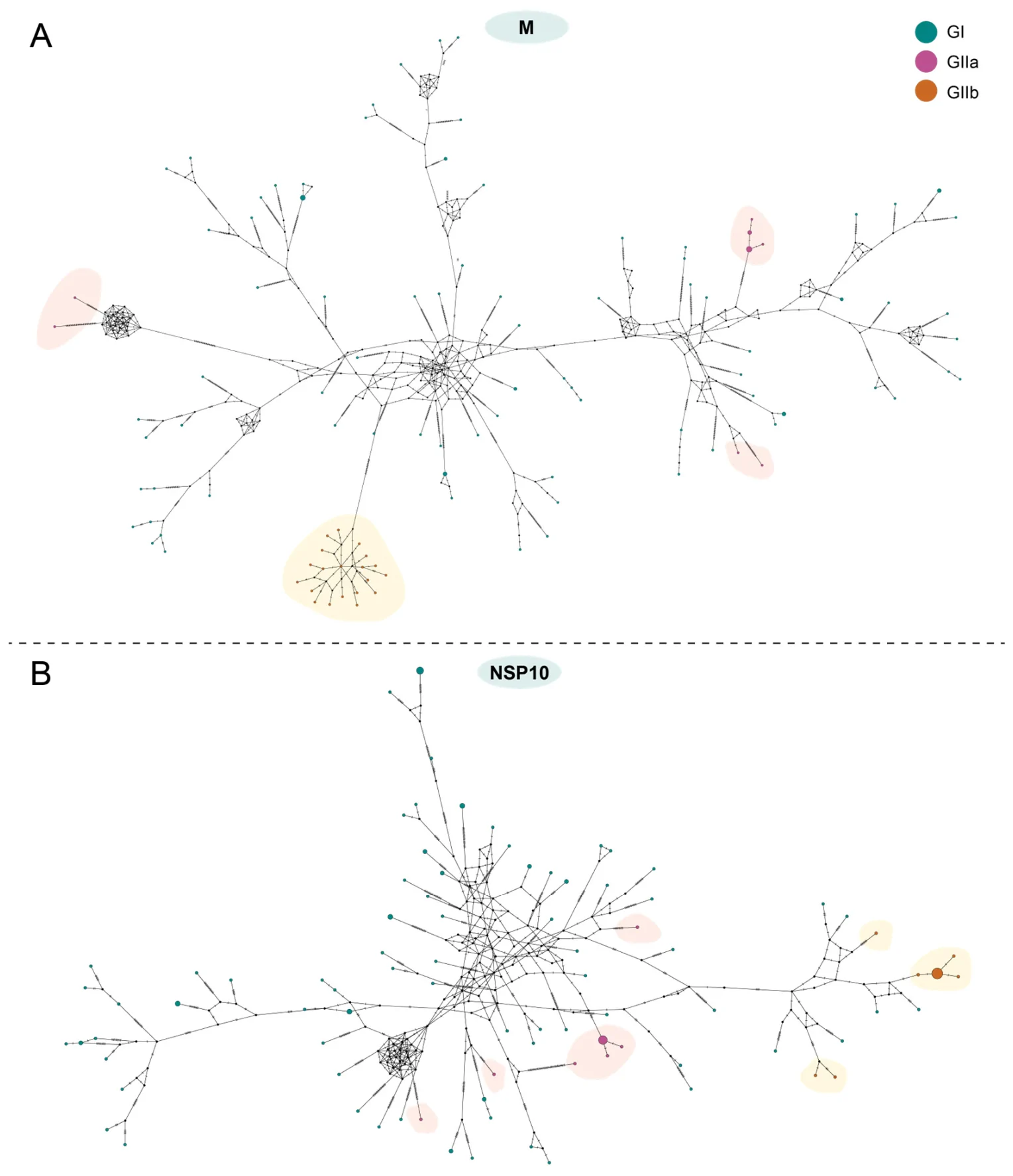
Haplotype networks of nonrecombinant FCoV genes. (**A**) Neighbor-joining haplotype network constructed from nucleotide sequences of the M gene. (**B**) Neighbor-joining haplotype network constructed from nucleotide sequences of the NSP10 gene. Both genes showed no detectable recombination signals in the recombination analyses. Each node represents a unique haplotype, with node size proportional to the number of FCoV sequences represented by that haplotype. Haplotypes derived from the GI, GIIa, and GIIb lineages are distinguished by different background colors. Connecting branches represent genetic relationships among haplotypes, and small tick marks on the branches indicate the number of nucleotide substitutions separating the connected haplotypes.

The M gene generated 107 haplotypes from 127 sequences, with no predominant haplotype identified, reflecting extensive sequence diversification driven by accumulated mutations (Figure 3A). Unlike the whole-genome phylogeny, the M gene haplotype network did not preserve the distinct lineage structure separating GI and GIIa. Instead, GI comprised numerous genetically divergent haplotypes distributed throughout the network, indicative of substantial within lineage diversity (Figure 3A). By comparison, the GIIb haplotypes formed a compact cluster with limited genetic divergence (Figure 3A). Despite the close sister relationship between GIIa and GIIb inferred from whole-genome phylogenetic analysis (Figure 1), the GIIb membrane haplotypes were more closely associated with a single GI haplotype than with those of GIIa (Figure 3A). Furthermore, the GIIa membrane haplotypes were partitioned into three genetically distinct groups separated by relatively large mutational distances, suggesting multiple evolutionary origins for this gene within the lineage (Figure 3A).

Analysis of the NSP10 gene produced 86 haplotypes from the same 127 genomes, again with no dominant haplotype, confirming a similarly high level of genetic diversity (Figure 3B). The GI NSP10 sequences were represented by numerous closely related haplotypes concentrated in the central region of the network, together with two more divergent peripheral clusters (Figure 3B). Consistent with the membrane gene, the GIIa NSP10 haplotypes were not recovered as a discrete lineage but were instead interspersed among the central GI associated haplotypes (Figure 3B). The GIIb NSP10 haplotypes likewise failed to form an independent lineage and were distributed among peripheral GI associated clusters while remaining genetically similar to one another (Figure 3B).

Notably, neither the membrane nor the NSP10 haplotype network recapitulated the close evolutionary relationship between GIIa and GIIb inferred from the whole-genome phylogeny. Instead, haplotypes from both lineages frequently showed closer relationships with GI than with each other. Collectively, these observations reveal substantial discordance between gene-level and genome-wide evolutionary relationships, consistent with the pervasive recombination identified across the FCoV genome.

### 3.4. Conserved Dinucleotide Composition Bias and Progressive CpG Depletion During FCoV Evolution

To characterize dinucleotide composition across FCoV genomes, the observed to expected ratio of each of the sixteen dinucleotides was calculated for every genome. In the absence of compositional bias, the observed to expected ratio is expected to approximate one.

**Figure 4:**
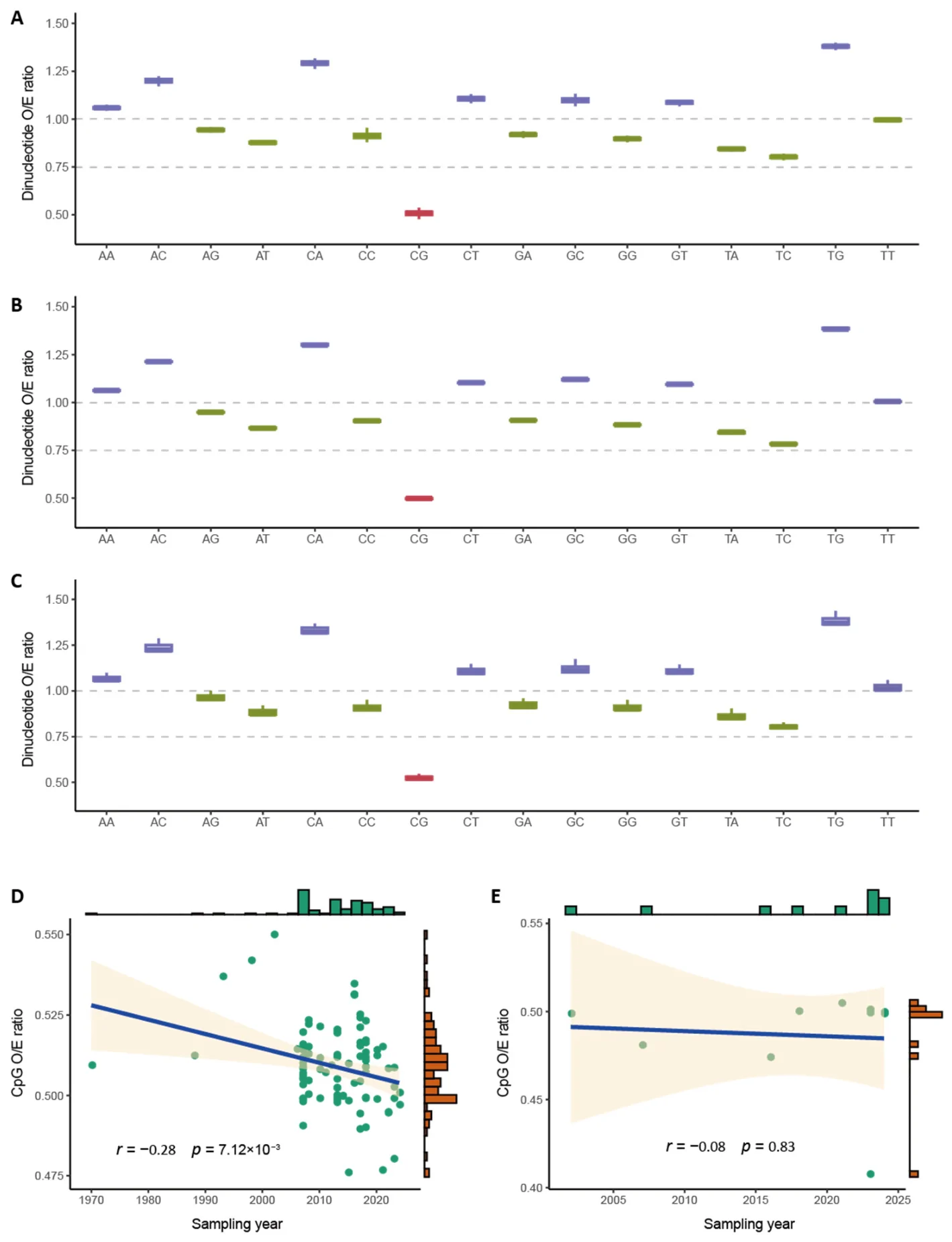
Dinucleotide composition bias and temporal dynamics of CpG abundance in FCoVs. (**A**–**C**) Observed-to-expected (O/E) ratios of the 16 possible dinucleotides in FCoV genomes belonging to GI (**A**), GIIa (**B**), and GIIb (**C**), respectively. Each boxplot represents the distribution of the corresponding dinucleotide O/E ratio across genomes within the indicated lineage. The horizontal reference line at O/E = 1 represents the expected value in the absence of dinucleotide compositional bias. Dinucleotides with O/E ratios greater than 1 are overrepresented, whereas those with ratios below 1 are underrepresented. (**D**) Temporal association between the CpG O/E ratio and sampling year among GI FCoV genomes. Each point represents an individual genome, and the fitted regression line indicates the temporal trend in CpG abundance. The association was evaluated using Pearson correlation analysis. (**E**) Temporal association between the CpG O/E ratio and sampling year among GIIa FCoV genomes. The relationship was evaluated using Pearson correlation analysis, with individual points representing sampled genomes and the fitted line indicating the temporal trend.

Pronounced dinucleotide bias was identified in the GI lineage. Seven dinucleotides were overrepresented with observed to expected ratios exceeding one, among which TpG exhibited the highest mean ratio of 1.38, followed by CpA at 1.29 and ApC at 1.2 (Figure 4A). In contrast, eight dinucleotides displayed observed to expected ratios between 0.75 and 1.00, indicating varying degrees of underrepresentation (Figure 4A). Although the ratio of TpT remained close to the expected value, TpC and TpA were consistently depleted. Several of these moderately underrepresented dinucleotides exhibited remarkably limited variation among GI genomes, as evidenced by the nearly overlapping interquartile ranges in the boxplots, suggesting that their relative frequencies have remained highly stable despite continuous sequence evolution.

Among all dinucleotides, CpG was consistently the most depleted, with a mean observed to expected ratio of 0.51 (Figure 4A), whereas TpG represented the most enriched dinucleotide. The marked contrast between CpG depletion and TpG enrichment highlights a pronounced imbalance in dinucleotide usage within FCoV genomes. Furthermore, CpG displayed greater variability among GI genomes than most other dinucleotides, indicating that the extent of CpG suppression differed among circulating strains.

The overall dinucleotide composition of GIIa closely resembled that of GI. Eight dinucleotides were overrepresented, with TpG, CpA, and ApC again exhibiting the highest observed to expected ratios of 1.39, 1.31, and 1.21, respectively (Figure 4B). CpG remained the most depleted dinucleotide with a mean ratio of 0.49 (Figure 4B). Compared with GI, however, dinucleotide frequencies within GIIa were considerably more homogeneous, with only limited variation detected among individual genomes.

A similar compositional profile was observed in GIIb. Approximately half of the dinucleotides were overrepresented, whereas the remainder were underrepresented. TpG, CpA, and ApC again represented the most enriched dinucleotides, with mean observed to expected ratios of 1.39, 1.34, and 1.24, respectively (Figure 4C). Consistent with both GI and GIIa, CpG exhibited the lowest observed to expected ratio at 0.53 (Figure 4C). Despite the recombinant origin of GIIa and GIIb, their dinucleotide composition remained highly similar to that of GI, indicating that the overall pattern of dinucleotide usage is conserved across alphacoronaviruses rather than being lineage-specific.

Because CpG consistently represented the most depleted dinucleotide in all three lineages, its temporal dynamics were further investigated. Within GI, the CpG observed to expected ratio exhibited a significant negative correlation with sampling year (*r* = −0.28, *p* = 7.12 × 10^−3^), demonstrating a gradual reduction in CpG frequency over time (Figure 4D). No significant temporal association was detected in GIIa (Figure 4E), whereas correlation analysis was not performed for GIIb because all available genomes were collected during the same year. Together, these findings demonstrate that CpG suppression represents a conserved genomic characteristic of FCoVs and that CpG depletion has continued throughout the evolutionary history of the GI lineage (Figure 4).

### 3.5. Distinct Selective Pressures Shape the Evolution of FCoV Genes

To characterize the selective pressures acting on FCoV genes, codon-based selection analyses were performed using FUBAR implemented in the Datamonkey platform. Collectively, these analyses revealed that positive selection was unevenly distributed across the FCoV genome and was concentrated in a subset of genes.

**Figure 5:**
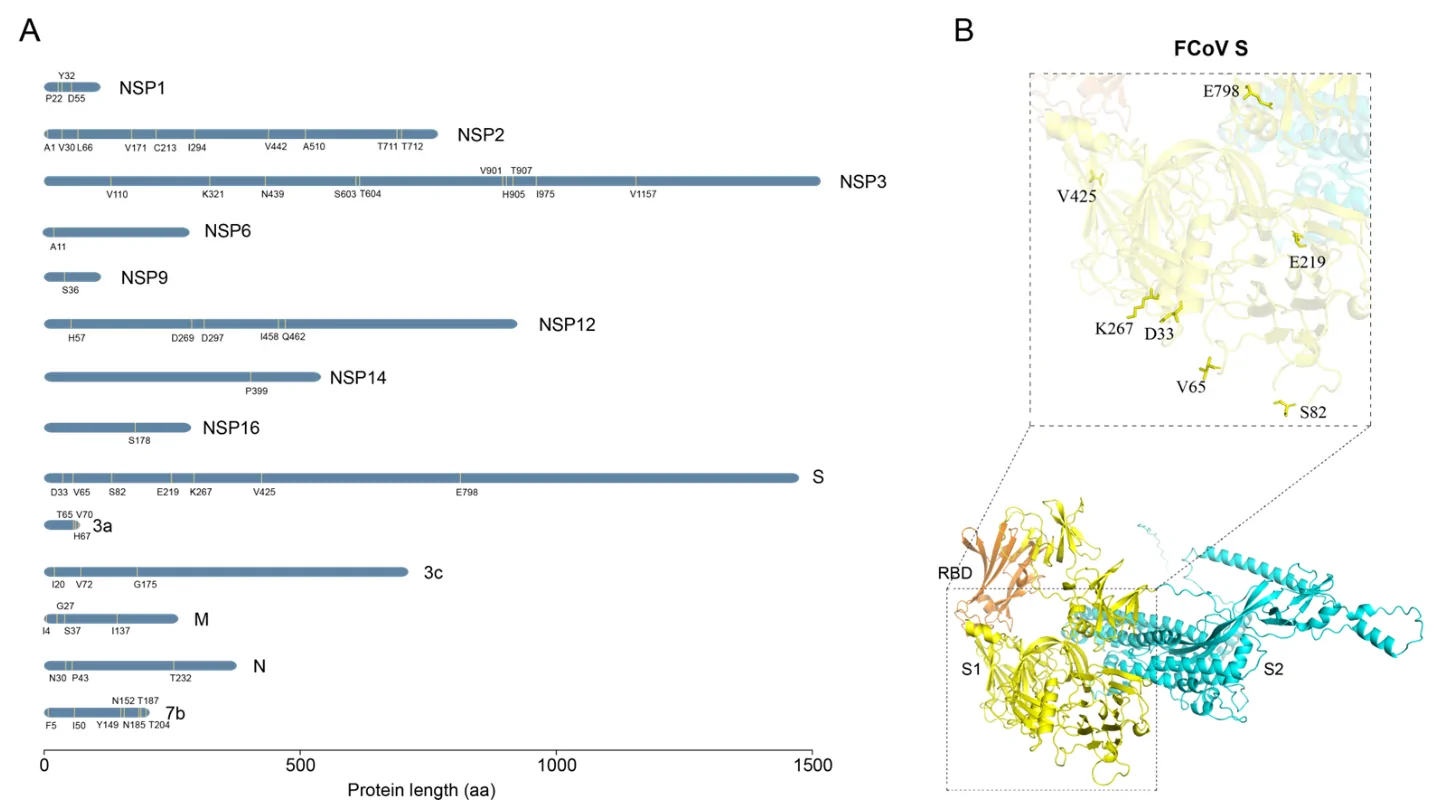
Heterogeneous patterns of positive selection across the FCoV genome. (**A**) Distribution of positively selected sites across the 14 FCoV genes identified by codon-based selection analyses. Each gene is represented by a horizontal bar scaled proportionally to its coding length, with the gene name indicated on the right. Positively selected sites identified by FUBAR are indicated by vertical marks positioned according to their amino acid locations. (**B**) Three-dimensional structure of the FCoV spike protein showing the positions of positively selected amino acid residues identified by FUBAR. Positively selected residues are mapped onto the protein structure and labeled according to their amino acid identity and position.

Among all genes, NSP2 and NSP3 exhibited the highest numbers of positively selected codon sites, with ten sites identified in each gene by FUBAR (Figure 5A). These results indicate that both NSP2 and NSP3 experienced substantial positive selection during FCoV evolution, highlighting these two genes as prominent targets of selective pressure within the FCoV genome.

For the S gene, the complete set of FCoV spike sequences was analyzed as a single dataset using FUBAR. Seven codon sites, corresponding to amino acid positions D33, V65, S82, E219, K267, V425, and E798, showed significant evidence of positive selection (Figure 5). These positively selected sites were distributed across the S1 domain of the spike protein, indicating that selective pressures were not restricted to a single functional region of the FCoV spike protein (Figure 5).

Positive selection was also detected in 11 additional FCoV genes by FUBAR (Figure 5A). Gene 7b contained the highest number of positively selected codon sites among these genes, with seven sites identified, followed by NSP12 and the M gene, with five and four sites, respectively. Three positively selected sites were identified in each of NSP1, the N gene, 3a, and 3c, whereas NSP6, NSP9, NSP14, and NSP16 each contained one positively selected site. Together, these results indicate that positively selected sites were distributed across multiple structural and nonstructural genes of the FCoV genome (Figure 5A).

Collectively, these findings demonstrate that positive selection has acted on a substantial proportion of FCoV genes during viral evolution. Notably, the genes showing the strongest signatures of positive selection largely overlapped with those exhibiting frequent recombination, identifying these genes as major evolutionary hotspots within the FCoV genome. The recurrent accumulation of adaptive substitutions in these genes is consistent with persistent evolutionary pressures imposed during virus host interactions.

## 4. Discussion

The present study provides a genome-wide perspective on the evolutionary dynamics of feline coronaviruses and indicates that FCoV diversification involves multiple evolutionary processes operating across different genomic scales. At the genome level, homologous recombination is associated with differences in genomic architecture and the emergence of distinct genetic lineages. At the gene level, sequence variation and episodic positive selection contribute to diversification of individual genomic regions. At a broader scale, the persistence of conserved genomic characteristics despite sequence variation suggests the presence of longer-term compositional constraints. Together, these findings highlight the coexistence of genomic diversification and evolutionary constraint during FCoV evolution.

One of the clearest manifestations of this hierarchical organization is the marked discordance between genome-wide and gene-level evolutionary relationships. Whole-genome phylogenetic analyses consistently resolved three well-supported lineages (Figure 1), whereas haplotype networks reconstructed from nonrecombinant genes failed to recover the same lineage relationships (Figure 3). Instead, genes from different genomic backgrounds frequently displayed closer relationships to ancestral GI viruses than to each other. Although phylogenetic incongruence is commonly regarded as evidence of recombination, our findings further demonstrate that it reflects a more fundamental characteristic of feline coronavirus evolution: different components of the viral genome may follow partially independent evolutionary trajectories. Consequently, the evolutionary history of a coronavirus genome cannot always be inferred from the history of individual genes.

This observation has broader implications for evolutionary studies of coronaviruses. Many previous investigations have relied on single genes, particularly the spike gene, to infer viral origins, transmission histories, or host adaptation because of its biological importance and extensive sequence availability [8,24]. However, coronaviruses evolve as integrated yet modular genomes. Individual genomic regions experience different combinations of recombination, mutation, and selective constraint, resulting in heterogeneous evolutionary histories across the genome. As a consequence, gene trees and genome trees should not be viewed as competing representations of viral evolution, but rather as complementary descriptions of evolutionary processes occurring at different biological scales [25]. In our dataset, the discordance between whole-genome phylogenetic relationships and gene-level haplotype patterns suggests that different genomic regions may retain partially distinct evolutionary histories. Thus, whole-genome and gene-based analyses may provide complementary perspectives on FCoV evolution, with whole-genome analyses reflecting relationships among complete genomic backgrounds and gene-level analyses highlighting variation within individual genomic regions.

Our results further suggest that mutation and recombination may play distinct evolutionary roles in feline coronavirus diversification. Extensive haplotype diversity within the membrane and NSP10 genes demonstrates that nucleotide substitutions accumulate continuously even in genomic regions lacking detectable recombination, indicating that mutational processes contribute to the generation of genetic diversity during long-term viral circulation (Figure 3). Nevertheless, the patterns of sequence variation observed in these genes alone do not fully recapitulate the major lineage relationships inferred from complete genomes. Instead, the genome-wide phylogenetic and recombination analyses indicate that recombination is associated with differences in genomic configuration among FCoV lineages. Taken together, these observations are consistent with complementary contributions of mutation and recombination to FCoV diversification, but do not establish their relative contributions or a specific temporal or causal relationship between the two processes.

The hierarchical framework proposed above further suggests that homologous recombination is not simply a mechanism for generating genetic diversity but a process that repeatedly reorganizes coronavirus genome architecture. Coronaviruses possess one of the highest homologous recombination frequencies among positive-sense RNA viruses because discontinuous RNA synthesis during replication facilitates frequent template switching by the viral RNA-dependent RNA polymerase [26,27]. As a consequence, extensive genomic fragments rather than individual nucleotides can be exchanged between closely related viruses within a single replication cycle. Compared with the gradual accumulation of point mutations, such large-scale genetic exchange enables FCoVs to explore novel genomic combinations on a substantially shorter evolutionary timescale.

Our analyses indicate that the emergence of the GIIa and GIIb lineages is consistent with repeated, rather than singular, recombination events between feline and canine coronaviruses. Although both lineages share a similar evolutionary origin involving CCoV-derived genomic regions, they possess distinct recombination architectures and breakpoint distributions, indicating that they were generated through independent evolutionary events instead of diversification from a common recombinant ancestor. Recurrent interspecies recombination has likewise been documented in several other alphacoronaviruses and betacoronaviruses, highlighting homologous recombination as a continual source of genomic innovation rather than an isolated historical occurrence [28,29]. Together, these observations suggest that the exchange of genetic material between related coronaviruses represents an ongoing evolutionary process capable of repeatedly generating novel viral genomic backgrounds.

An important implication of these findings is that feline coronavirus genomes appear to evolve as assemblies of interacting functional modules rather than as indivisible genetic units. The independent replacement of genomic regions encompassing the spike gene and portions of ORF1b in the recombinant lineages indicates that certain genomic regions can be exchanged while preserving the functionality of the remaining genome. Similar patterns have been observed in other coronaviruses, where genes associated with host interaction frequently exhibit evolutionary histories distinct from those of the core replication machinery [30,31]. Such modularity likely increases evolutionary flexibility by allowing individual functional components to be replaced or optimized without requiring simultaneous modification of the entire genome. However, the modular nature of FCoV genomes should not be interpreted as unlimited interchangeability. Functional modules remain embedded within highly coordinated replication networks, and successful recombination ultimately depends on the compatibility between newly acquired genomic regions and the pre-existing genetic background.

If recombination generates new genomic architectures, an equally important question is why evolutionary change is repeatedly concentrated within only a subset of viral genes. Our analyses revealed a striking overlap between genes exhibiting frequent recombination and those undergoing recurrent positive selection, particularly NSP3, the spike gene, NSP2, NSP12, and 7b gene (Figure 5). This overlap indicates that some genomic regions experience multiple forms of evolutionary variation, but does not establish a direct relationship between recombination and positive selection. In other words, evolutionary innovation in FCoVs appears to be focused within a limited number of functional modules that mediate critical interactions between the virus and its host.

This interpretation is consistent with a growing body of evidence indicating that FCoV evolution is highly heterogeneous across the genome. Viral proteins differ substantially in the degree to which they interact with host molecules, immune defenses, and changing cellular environments. Proteins positioned at the virus–host interface are expected to experience continual selective pressure as viruses optimize replication efficiency while simultaneously evading host antiviral responses [32]. Under such conditions, any genetic change capable of improving viral fitness—whether introduced through mutation or recombination—has an increased probability of being retained by natural selection. Conversely, proteins performing highly conserved structural or enzymatic functions are subject to stronger purifying selection because even minor sequence alterations may compromise essential viral processes. The heterogeneous evolutionary landscape observed across the FCoV genome therefore likely reflects differences in functional constraint rather than differences in intrinsic mutation or recombination rates.

Among all genes, NSP3 represents the clearest example of this evolutionary principle. NSP3 not only exhibited the strongest evidence of recombination but also contained the largest number of positively selected codons identified in this study. This multifunctional protein occupies a central position within the coronavirus replication cycle, integrating proteolytic processing, replication complex assembly, membrane remodeling, and multiple mechanisms of innate immune antagonism [33]. Unlike proteins dedicated to a single biological function, NSP3 is continuously exposed to selective pressures arising from both viral replication and host antiviral defenses. Its exceptional evolutionary dynamics therefore likely reflect the convergence of multiple selective forces acting simultaneously on a single protein.

The evolutionary pattern of the spike protein illustrates a complementary but distinct adaptive strategy. Whereas NSP3 primarily mediates intracellular interactions with the host, the spike glycoprotein defines the extracellular interface through which coronaviruses recognize host receptors and encounter antibody-mediated immune pressure. It is therefore unsurprising that positively selected sites were concentrated within the S1 subunit, where receptor-binding and antigenic determinants are located, while the membrane-fusion machinery within S2 remained comparatively conserved. Notably, both independent recombinant lineages identified in this study involved replacement of the spike gene, indicating that modification of host interaction modules has repeatedly accompanied lineage emergence during FCoV evolution.

Adaptive evolution was also detected in NSP2, NSP12, and the nucleocapsid protein, highlighting that coronavirus adaptation extends well beyond receptor recognition (Figure 5A). Although these proteins perform diverse biological functions, they all contribute to processes that directly influence viral fitness within infected cells, including regulation of replication, genome synthesis, intracellular organization, and modulation of host responses [34]. Their adaptive diversification therefore reinforces the view that successful FCoV evolution depends on coordinated optimization of multiple components of the viral life cycle rather than modification of any single protein. Collectively, these findings suggest that adaptive evolution in FCoVs is distributed across an interconnected functional network, with individual proteins evolving under different selective regimes but contributing collectively to viral adaptation.

Importantly, the coexistence of recurrent recombination and positive selection within the same genes should not be interpreted as evidence that one process directly drives the other. Instead, both processes are likely consequences of the same underlying evolutionary principle: genes experiencing the strongest interactions with the host are simultaneously the genes in which evolutionary innovation is most strongly favored. Recombination provides access to alternative genetic variants, whereas positive selection determines which of these variants, together with newly arising mutations, confer sufficient fitness advantages to persist during long-term viral circulation. This overlap indicates that some genomic regions experience multiple forms of evolutionary variation, but does not establish a direct relationship between recombination and positive selection.

The evolutionary patterns described above indicate that extensive genomic innovation in FCoVs is accompanied by equally pervasive evolutionary constraint. While recombination repeatedly reorganizes genome architecture and adaptive evolution continuously modifies individual proteins, several fundamental genomic characteristics remain remarkably stable throughout viral diversification. This apparent paradox highlights an important feature of FCoV evolution: successful adaptation does not require unrestricted genetic change but instead occurs within boundaries imposed by long-term functional and genomic constraints [30].

The highly conserved dinucleotide composition observed across all three evolutionary lineages exemplifies these constraints. Despite their independent evolutionary histories and distinct recombinant architectures, GI, GIIa, and GIIb retained nearly identical patterns of dinucleotide usage, characterized by enrichment of TpG and CpA together with persistent depletion of CpG (Figure 4). These observations indicate that large-scale genomic exchange alone is insufficient to fundamentally alter the compositional properties of FCoV genomes. Instead, genome composition appears to remain remarkably resilient despite continual turnover of primary nucleotide sequence, implying that compositional evolution operates under constraints that extend beyond the inheritance of individual genomic regions.

Among these conserved features, CpG depletion represents one of the most prominent genomic signatures of vertebrate RNA viruses. Although reduced CpG frequencies are widely interpreted as evidence of adaptation to host antiviral mechanisms, particularly ZAP-mediated recognition of CpG-rich viral RNA, increasing evidence suggests that no single mechanism adequately explains this phenomenon [35,36]. Mutational biases, codon usage, RNA structural requirements, replication-associated constraints, and multiple host selective pressures are all likely to contribute to long-term CpG evolution. The gradual reduction in CpG observed within the GI lineage is therefore consistent with long-term compositional change, but should be interpreted cautiously because the available sequences are unevenly distributed across sampling years and geographic regions. In particular, temporal and geographic effects cannot be completely disentangled in the present dataset, and the observed negative correlation between CpG abundance and sampling year does not by itself establish a purely temporal effect. Nevertheless, the observed pattern is compatible with the possibility that CpG composition has gradually changed during the diversification of GI FCoVs. The absence of a similar temporal trend in GIIa should be interpreted cautiously, given its more recent origin, narrower sampling period, and recombinant evolutionary history.

The pronounced and persistent CpG depletion observed in FCoVs may also have implications beyond understanding their evolutionary history. In other RNA viruses, including influenza A virus, experimentally increasing synonymous CpG dinucleotides has been shown to attenuate viral replication while preserving the encoded protein sequences, highlighting CpG manipulation as a potential strategy for rational attenuation [37,38]. Whether a similar approach could be exploited for FCoVs remains unknown, but the conserved CpG constraint identified here provides an evolutionary rationale for investigating synonymous CpG modification as a potential route toward rational FCoV attenuation.

Whether these genomic evolutionary patterns are associated with the transition from FCoV infection to FIP remains an important but unresolved question, as the heterogeneous phenotypic information available for the present dataset does not allow a systematic phenotype-stratified analysis. Notably, previous work has linked specific recombinant FCoV–CCoV lineages to FIP outbreaks, highlighting the potential relevance of genomic recombination to pathogenic phenotypes [7].

Taken together, our findings support a conceptual framework for FCoV evolution in which genomic diversification occurs alongside evolutionary constraints. The patterns observed in our dataset indicate that recombination is associated with variation in genomic architecture, whereas mutation and positive selection contribute to sequence diversification across individual genes. At the same time, the persistence of conserved genomic characteristics despite sequence variation suggests the influence of longer-term genomic constraints. Although the relative contributions and temporal relationships among these processes were not quantitatively assessed in the present study, their contrasting patterns across genomic scales provide a framework for interpreting FCoV diversification as a balance between genomic plasticity and evolutionary constraint. Further comparative and experimental studies will be required to determine how these processes influence viral fitness, host adaptation, and pathogenic phenotypes.

## 5. Conclusions

Our findings reveal that the long-term evolution of feline coronaviruses is shaped by a dynamic balance between genomic innovation and evolutionary constraint. Recurrent recombination, including independent exchanges with canine coronaviruses, contributes to the emergence of distinct genomic lineages, whereas mutation and positive selection drive continued diversification within individual genes. These processes are particularly concentrated in evolutionary hotspots such as NSP3 and S, highlighting the importance of both replication-associated and host-interacting proteins in FCoV adaptation. At the same time, the persistent depletion of CpG across major lineages and its progressive decline over time indicate that broader genomic constraints remain conserved despite extensive sequence turnover and recombination. Together, these findings support a conceptual framework in which FCoV diversification involves both genomic plasticity and evolutionary constraint, rather than a single dominant evolutionary process. Although the relative contributions and temporal relationships among recombination, mutation, and selection were not quantitatively assessed in this study, their contrasting patterns across genomic scales provide new insights into FCoV diversification and may inform future evolutionary surveillance of feline and related alphacoronaviruses.

## Data Availability

The original contributions presented in this study are included in the article material. Further inquiries can be directed to the corresponding authors.

## Supplementary Materials

The following supporting information can be downloaded at: https://www.mdpi.com/article/10.3390/v18091037/s1, Table S1: Information on the FCoV genome sequences analyzed in this study.

## Abbreviations

The following abbreviations are used in this manuscript:

FCoV	feline coronavirus
FIPV	feline infectious peritonitis virus
CCoV	canine coronavirus
