# Supplementary material for: Balancing Genome Plasticity and Evolutionary Constraint During the Long-Term Evolution of Feline Coronaviruses
Source: Viruses. 2026 Sep 19;18(9):1037. doi: 10.3390/v18091037 (PMC13612032; doi:10.3390/v18091037)
Supplement: Supplementary file 1 [file viruses-18-01037-s001.zip › viruses-4539859-supplementary.pdf]

Supplementary Table S1. Information on the FCoV genome sequences analyzed in this study.

Accession Number	Sampling year	Geographic origin
EU186072.1	1970	USA
FJ938051.1	2002	USA
FJ938052.1	2007	NLD
FJ938053.1	2007	NLD
FJ938054.1	2007	NLD
FJ938055.1	2007	NLD
FJ938056.1	2007	NLD
FJ938057.1	2007	NLD
FJ938058.1	2007	NLD
FJ938059.1	2007	NLD
FJ938060.1	1993	USA
FJ938061.1	1998	USA
FJ938062.1	2007	NLD
GQ152141.1	2007	CHN
GU553361.1	2007	NLD
GU553362.1	2007	NLD
HQ012367.1	2007	NLD
HQ012368.1	2007	NLD
HQ012369.1	2007	NLD
HQ012370.1	2008	NLD
HQ012371.1	2008	NLD
HQ012372.1	2007	NLD
HQ392469.1	2008	NLD
HQ392470.1	2007	NLD
HQ392471.1	2007	NLD
HQ392472.1	2008	NLD
JN183882.1	2010	NLD
JN183883.1	2010	NLD
KF530123.1	2010	NLD
KF530130.1	2010	NLD
KP143507.1	2013	GBR
KP143508.1	2013	GBR
KP143509.1	2013	GBR
KP143510.1	2013	GBR
KP143511.1	2013	GBR
KP143512.1	2013	GBR
KX722529.1	2015	BEL
KX722530.1	2015	DNK
KY292377.1	2016	CHN
KY566209.1	2016	CHN
KY566210.1	2016	CHN

KY566211.1	2016	CHN
LC742526.1	2015	JPN
LC852817.1	2024	JPN
LC880185.1	1988	JPN
MG893511.1	2012	DEU
MN165107.1	2018	CHN
MT239439.1	2016	CHN
MT239440.1	2017	CHN
MT444152.1	2020	CHN
MW030108.1	2018	CHN
MW030109.1	2018	USA
MW030110.1	2018	CHN
MW308128.1	2006	DEU
ON595853.1	2017	AUS
ON595854.1	2017	AUS
ON595855.1	2017	AUS
ON595856.1	2017	AUS
ON595857.1	2017	AUS
ON595858.1	2017	AUS
ON595862.1	2018	AUS
ON595863.1	2018	AUS
ON595866.1	2018	AUS
ON595867.1	2018	AUS
ON595868.1	2018	AUS
ON595869.1	2018	AUS
ON595870.1	2018	AUS
ON595871.1	2018	AUS
OP542206.1	2020	USA
OR295209.1	2021	CHN
OR711916.1	2023	CHN
PP254207.1	2022	IND
PP854701.1	2008	USA
PP854702.1	2008	USA
PP908788.1	2021	THA
PP908789.1	2021	THA
PQ133176	2023	CYP
PQ133177	2023	CYP
PQ133178	2023	CYP
PQ133179	2023	CYP
PQ133180	2023	CYP
PQ133181	2023	CYP
PQ133182	2023	CYP
PQ133183	2023	CYP
PQ133184	2023	CYP

PQ133185	2023	CYP
PQ133186	2023	CYP
PQ133187	2023	CYP
PQ133188	2023	CYP
PQ133189	2023	CYP
PQ133190	2023	CYP
PQ133191	2023	CYP
PQ133192	2023	CYP
PQ133193	2023	CYP
PQ133194	2023	CYP
PQ133195	2023	CYP
PQ192636.1	2024	USA
PQ202302.1	2024	USA
PQ416775.1	2023	CHN
PV289782.1	2024	CHN
PV780394.1	2023	CHN
PV780395.1	2023	CHN
PV797400.1	2022	THA
PV797401.1	2022	THA
PV797402.1	2023	THA
PV797403.1	2023	THA
PV941793.1	2002	USA
PX132896.1	2013	USA
PX315748.1	2011	GBR
PX315749.1	2013	GBR
PX315750.1	2013	GBR
PX315751.1	2013	GBR
PX315752.1	2013	GBR
PX315753.1	2013	GBR
PX315754.1	2013	GBR
PX315755.1	2014	GBR
PX315756.1	2014	GBR
PX315757.1	2017	GBR
PX315758.1	2017	GBR
PX471051.1	2023	CHN
PZ043810.1	2020	CHN
AY994055.1	/	USA
NC_002306.3	/	USA
DQ848678.1	/	/
JN634064.1	/	/
JQ408980.1	/	/
JQ408981.1	/	/
